# The proteus effect on human pain perception through avatar muscularity and gender factors

**DOI:** 10.1038/s41598-024-61409-4

**Published:** 2024-05-23

**Authors:** Youchan Yim, Zongheng Xia, Yuki Kubota, Fumihide Tanaka

**Affiliations:** University of Tsukuba, Tsukuba, 305-8573 Japan

**Keywords:** Information technology, Computer science

## Abstract

The Proteus effect, which occurs when using an avatar in virtual reality, influences user behavior, changes attitudes, and improves physical performance. Here, we show that human pain perception can be alleviated by the Proteus effect. To investigate the pain alleviation effect of using an avatar in a virtual environment, we conducted two experiments using a head-mounted display and a thermal pain stimulator to induce acute pain. The first experiment involved 20 adult participants, while the second experiment involved 44 adult participants. Experimental results show that participants reported significantly lower pain scores (15.982% reduction), as measured by the Pain Assessment Scale (PAS), when using a muscular avatar than when using a normal avatar. The experiments also revealed several significant gender factors. For example, participants reported significantly lower pain scores when using a gender-congruent avatar. In addition, the use of a muscular avatar was particularly effective for male participants. In contrast, female participants consistently reported lower pain scores when using the avatar regardless of its body type (muscular/normal). To further our understanding, we also measured participants’ gender-related pain stereotypes using the Gender Role Expectations of Pain (GREP) questionnaire, as well as participants’ sense of embodiment. The results of these questionnaires are consistent with the results of the PAS, suggesting possible relationships between stereotypes and the Proteus effect on pain perception, and between the degree of immersion in an avatar and the user’s perception of pain.

## Introduction

Distracting patients’ attention has been recognized as an effective method to alleviate the pain, distress, and anxiety induced by medical procedures^1–4^. Among the various media used to distract attention, virtual reality (VR), with its characteristic of high immersion^5^, offers a more effective alternative to traditional methods in dispersing focused attention on pain perception. For example, it has been found that a higher level of immersion in a VR environment significantly reduces the pain level of thermal stimuli^6–8^. Another study reported that co-administration of VR therapy with conventional analgesics in emergency rooms significantly alleviated pain^9^. While the immersive effects of VR offer significant advantages in the medical field, there is also growing interest in research that exploits the powerful interaction between vision and somatosensory perception^10,11^. In particular, studies using virtual avatars within VR environments show promising developments^12,13^. This approach not only exploits the immersive quality of VR, but also explores the intricate dynamics of embodying a virtual self, adding a new dimension to pain management and patient experience. Recent studies use avatars that are better synchronized with the user’s movements^14^ and integrate tactile feedback^15^, thereby increasing the user’s immersion in the VR environment.

On the other hand, as immersion increases, VR research has reported an interesting effect known as the Proteus effect^16–19^. The Proteus effect is a phenomenon based on research that suggests that when users see themselves reflected in an avatar in VR, their perceptions and behaviors change according to the characteristics of the avatar^16–20^. For instance, when an avatar appears strong and robust, users tend to perceive themselves as more resilient, and there is evidence that embodying avatars with these characteristics can lead to improved physical performance or even alleviate chronic pain^21–24^. In this paper, we explore this Proteus effect for pain management. A deep connection with the avatar, combined with an altered perception based on the avatar’s characteristics, may help to divert attention from the perception of pain. In particular, when users perceive themselves through an avatar that appears to be more resilient or less sensitive to pain, their actual pain perception may also decrease. However, no study has examined how these avatar characteristics might affect pain perception in healthy adults exposed to acute pain.

This paper reports the results of two experiments conducted to investigate whether the Proteus effect induces a change in the perceived pain intensity of human participants. Specifically, the following hypotheses were tested: (H1) the use of an avatar in a VR environment will decrease the user’s perceived pain intensity (the user will report less pain) compared to a real-world baseline condition, and (H2) the use of a muscular avatar will decrease the user’s perceived pain intensity compared to the use of a normal avatar. To this end, we first tested two types (muscular/normal) of male VR avatars by comparing them to a control condition in which participants recognized themselves from a real mirror. However, this first experiment made us aware of the crucial role of gender factors, such as the gender of the participant and the gender of the avatar. We then conducted the second experiment with these gender factors to thoroughly test the above hypotheses.

## Methods

### Participants

20 healthy Chinese and Japanese students participated in the first experiment, and 44 healthy Chinese students participated in the second experiment (see Supplementary Table S1). They were all recruited at the University of Tsukuba, Japan. None of the participants suffered from acute or chronic pain, bruises on the arms, skin diseases, or mental diseases such as depression and communication disorders. Each participant was compensated with 1380 JPY. The research protocols for both experiments were approved by the Research Ethics Committee of the Faculty of Engineering, Information, and Systems in the University of Tsukuba (2021R482 and 2022R711). All participants provided written informed consent, and all methods were carried out in accordance with the relevant guidelines and regulations.

### Study design

In the first experiment, participants were divided into two groups based on the avatar body type (ABT: muscular/normal). In each group, participants compared the avatar condition to a control condition in which participants did not use the avatar and recognized themselves from a real mirror (Environment: real/virtual).

The second experiment tested H2 in more detail with gender factors: the gender of the participant (GoP: male/female) and the gender of the avatar (GoA: male/female). Participants were divided into four groups according to ABT (muscular/normal) and GoP (male/female).

### Equipment

#### Virtual avatars

**Figure 1:**
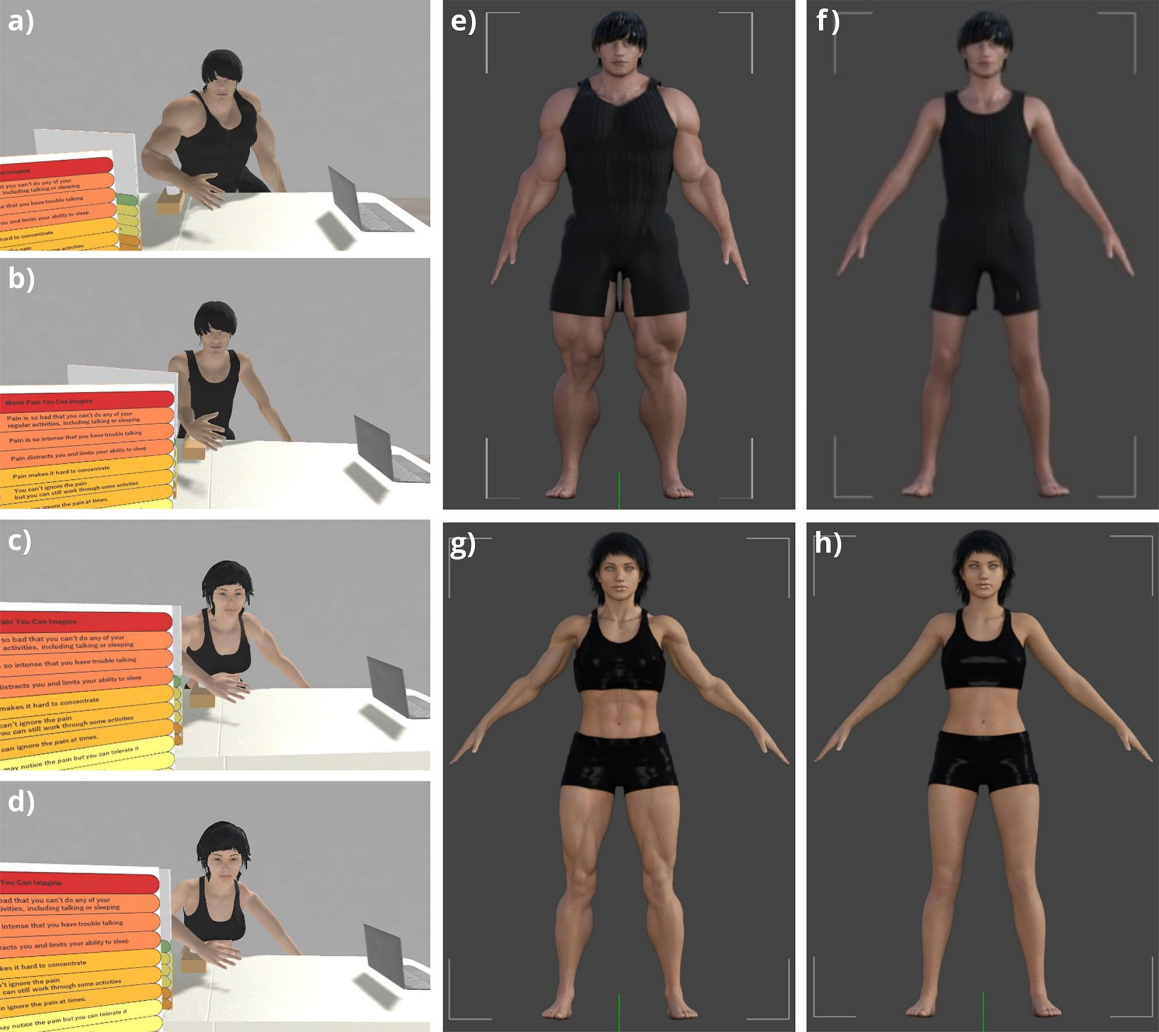
The avatars used in this study were created using the Daz3D software (Version 4.21)^25^ and rendered in Unity 2019 (Version 2019.4.18f1). The subfigures (**a**–**d**) show views from a head-mounted display (HMD), representing different avatar perspectives experienced by the participants: (**a**) male-muscular, (**b**) male-normal, (**c**) female-muscular, (**d**) female-normal. The subfigures (**e**–**h**) show full body shots of these avatars: (**e**) male-muscular, (**f**) male-normal, (**g**) female-muscular, (**h**) female-normal.

**Figure 2:**
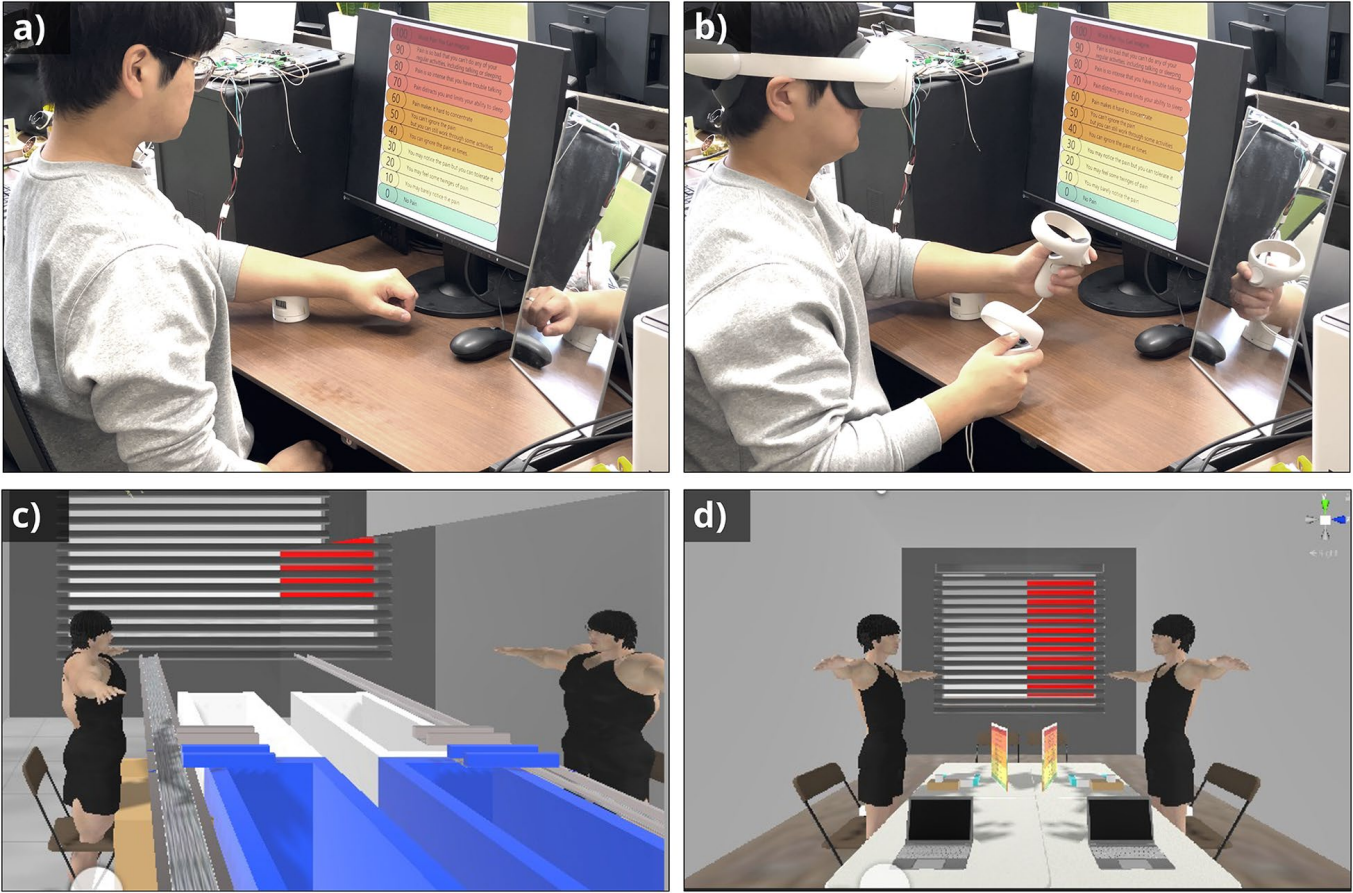
The experimental environment: (**a**) a participant receiving pain stimuli in the real environment of the first experiment, with a mirror positioned diagonally in front of the participant on the right for the 60-s familiarization. (**b**) a participant receiving pain stimuli while using a VR avatar in both the first and second experiments. (**c**) the appearance of the avatar in the virtual environment during the self-familiarization phase, where the avatar is reflected in a virtual mirror synchronized with the participant’s movements. This phase includes a task of sorting colored balls on a conveyor belt into corresponding baskets while listening to white noise, lasting 60 seconds in the first experiment and 100 s in the second experiment, designed to facilitate adaptation to the VR environment and avatar embodiment. (**d**) the virtual environment setup for participants receiving pain stimuli while using a virtual avatar in both experiments, with elements included to mimic the real world setting and facilitate PAS score responses.

Figure 1 shows the four avatars used in this study. In the first experiment, only male avatars were used, while in the second experiment, all four avatars were used. The virtual environment (Fig. 2) in our experiment was developed using Unity (Version 2019.4.38f1), and the avatar designs were created using Daz3D^25^ software (Version 4.21). We adjusted the body data of the humanoid avatars using Genesis 8 Male and Female Body Morphs, and for facial features, clothing, and other details, MASSIVE Morphs for Genesis 8 Male and Female were used under the Interactive License. Both the male and female avatars were designed based on the average height of Chinese adults born in 1996^26^. Body fat percentages were modeled based on average values for typical individuals (20–22%) and bodybuilders (6–8%). The muscular body type avatars were intended to represent individuals who exercise regularly, showing robustness and health, while the normal body type avatars represented an average body size.

In both experiments, participants were synchronized with avatars using a head-mounted display (HMD). Our virtual avatar mounting system used a Dell XPS 8940 PC and a Meta Quest 2. The mounting process involved capturing the physical location and movements of the user and then synchronizing them with the movements of the virtual avatar. The HMD and control handles were used to capture the user’s head and hand position information. Movement synchronization between the human and the avatar was achieved using the VRIK (Final IK) program, which sets 22 body position reference points, including the head, neck, and shoulder joints. This information was then projected back onto the HMD, allowing synchronized activities between the user and the avatar. Figure 2 shows the environment used in this study.

#### Thermal pain stimulator

**Figure 3:**
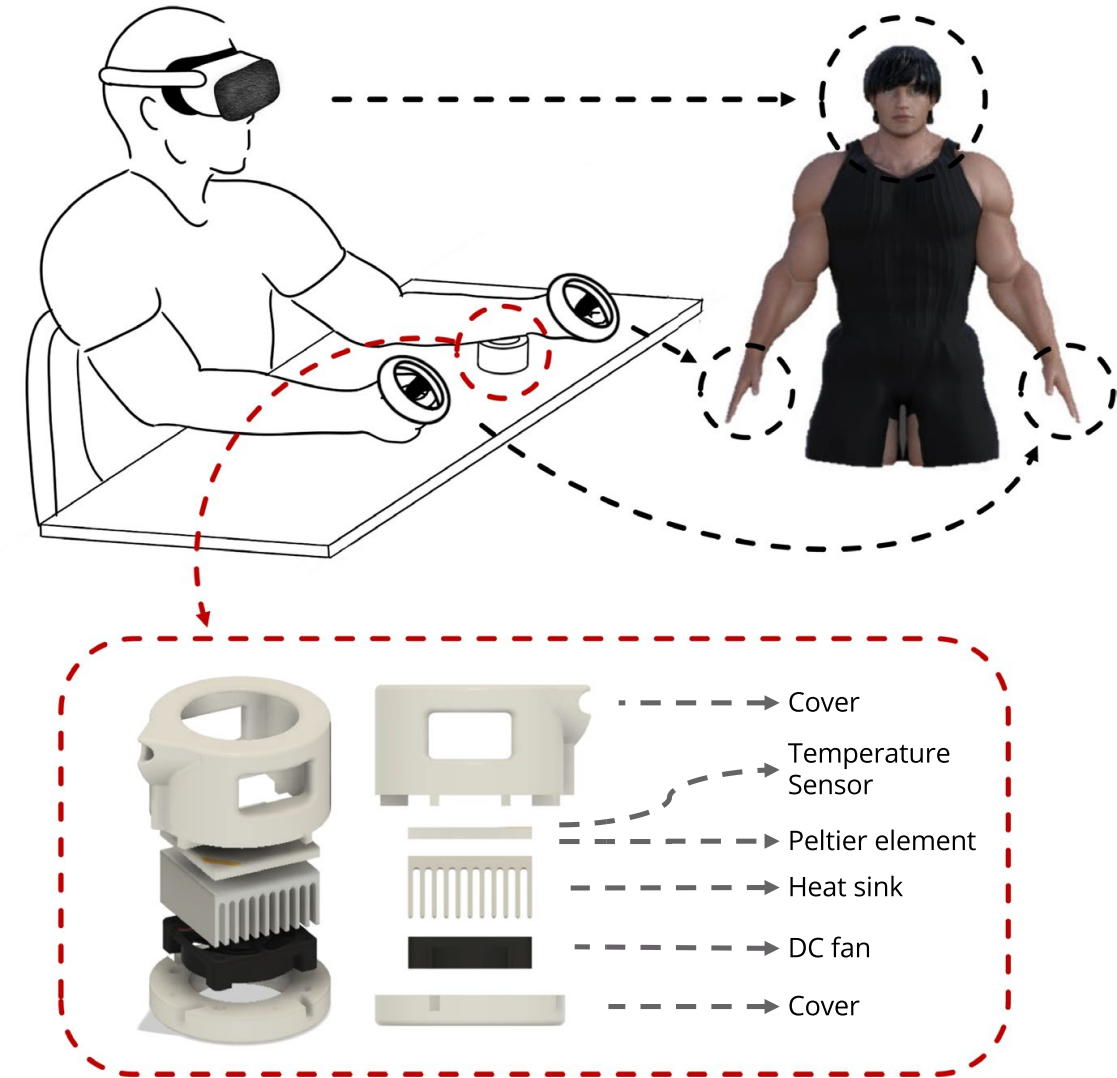
Illustration of the experimental setup. The participant is equipped with the Meta Quest 2 VR headset and holds the corresponding controllers in both hands. In the VR environment, activities are synchronized with the avatar based on the participant’s head position (determined by the position of the VR headset) and the positions of both hands (tracked by the controllers). During the main session, participants place the inside of their left forearm on a thermal stimulation device on a table through which they receive the pain stimulus.

In this study, we employed a pain research protocol using thermal stimulation^27^ (Fig. 3). A thermal stimulator with a 30 mm diameter contact probe was used. Its temperature was controlled by a Peltier element drive temperature controller (PLC-24V10A, Kurag Electronics, Tokyo, Japan) capable of PID temperature control. The device detected the temperature in real time using a temperature sensor (103JT-025, SEMITEC Corporation, Tokyo, Japan). Following previous studies^27,28^, participants were instructed to touch the skin around the elbow joint of their non-dominant arm to the contact probe of the thermal stimulator (see Figs. 2 and  3). The temperature of the thermal stimulation was either ${46}\,^\circ \textrm{C}$ or ${45}\,^\circ \textrm{C}$, which was determined in pilot experiments to correspond to the Pain-60 level^29–31^.

### Measurements

#### PAS

Subjective pain ratings were measured using the Allina Health Pain Assessment Scale (PAS)^32^. While the original scale uses a scale of 0–10, where 0: no pain, 1–3: mild pain, 4–6: moderate pain, and 7–10: severe pain, we adopted a modified version scaled by a factor of 10, resulting in a range of 0–100 (Supplementary Fig. S1), in line with previous pain research^27–30^. Participants verbally reported their pain rating immediately after the 40-s pain stimulus.

#### GREP

To measure gender-related pain stereotypes and perceptions based on avatar muscularity, we used a modified version of the Gender Role Expectations of Pain (GREP) questionnaire^33–35^. Details of the modified items can be found in Supplementary Table S2. This modified GREP questionnaire included 15 items in three domains: Sensitivity to Pain, Endurance of Pain, and Willingness to Report Pain. Participants rated each item on a scale of 0–100 in 10-point increments. The GREP was part of the pre-experimental questionnaire administered during the preliminary session and was only measured in the second experiment.

#### The sense of embodiment

The sense of embodiment in the virtual environment was assessed using a specific questionnaire^36,37^. This questionnaire contained 13 items that were scored on a 7-point Likert scale in four categories: body ownership, response, appearance, and multi-sensory. Detailed items for this questionnaire can be found in Supplementary Table S3. The average score of these four categories was used to calculate the embodiment score. This questionnaire was administered after the thermal stimulation.

### Procedure

**Figure 4:**
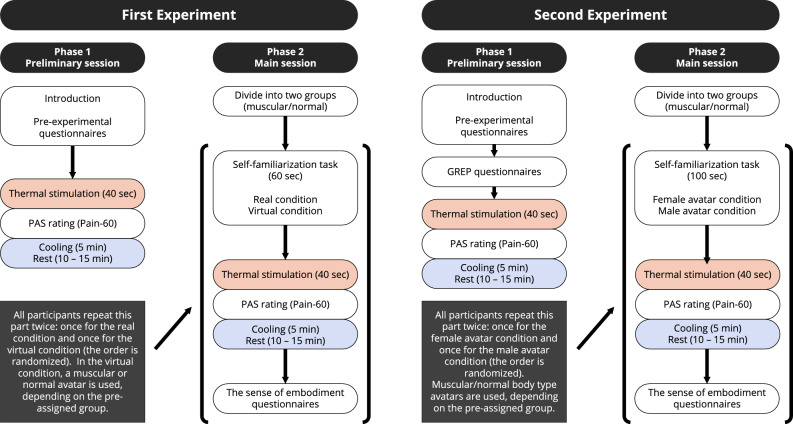
Flow charts of the first experiment (left) and the second experiment (right).

The basic procedures and settings of the two experiments were the same. However, in order to test gender factors, the second experiment included distinct variations such as the introduction of female avatars and sessions. In addition, GREP and sense of embodiment were only measured in the second experiment. The flow charts of the two experiments are shown in Fig. 4. The allocation of all participants in both experiments is summarized in Supplementary Table S1. The experiments were conducted in a controlled environment at a temperature of ${23}\,^\circ \textrm{C}$.

#### First experiment

The experimental procedure consisted of two phases. In Phase 1, participants were first informed of the purpose of the experiment, the methodology, the pain induction process, and the ethical considerations. Participants then completed pre-experimental questionnaires to collect demographic data, including age, gender, and VR experience. Phase 1 was a preliminary session in which participants experienced thermal pain stimuli and were familiarized with the basic procedure by undergoing a baseline measurement session in which a ${45}\,^\circ \textrm{C}$ thermal stimulus was applied for 40 s and then a PAS rating was made. If this temperature matched the participant’s Pain-60 level^29–31^, it was retained for the main session. Otherwise, a ${46}\,^\circ \textrm{C}$ thermal stimulus was tested and selected if appropriate. After each thermal stimulus, participants cooled the affected forearm area with an ice pack for 3 min, followed by a 2-min rest.

After an additional rest period of 10–15 min, Phase 2 (the main session) began. Participants were randomly assigned to either the normal or the muscular avatar group, with care taken to ensure a balanced gender distribution between these groups (see Supplementary Table S1). All participants then performed two environments (real/virtual) in a randomized order. In the real environment, participants performed a 60-s task to observe and recognize their own movements using a physical mirror. In the virtual environment, participants wore an HMD and performed a 60-s self-familiarization task in a VR environment using a virtual mirror (see Fig. 2). Thermal stimulation (40 s) was then applied at the predetermined temperature, after which participants rated their pain using the PAS as in Phase 1. After removing the HMD, participants underwent the cooling process and completed the post-stimulation questionnaires. The sense of embodiment and avatar evaluation questionnaires were administered exclusively in the virtual environment. After a 10-min break, participants completed these procedures again in the alternative environment.

#### Second experiment

The experimental procedure of the second experiment also consisted of two phases. Phase 1 of the second experiment was identical to that of the first experiment, except for the additional measurement of GREP that was administered during the pre-experimental questionnaires.

In Phase 2, participants in the second experiment were also randomly assigned to either the normal or the muscular avatar group, with care taken to ensure a balanced gender distribution between these groups, ensuring twice the population size of the first experiment to test gender factors (see Supplementary Table S1). In the second experiment, participants performed two virtual conditions (male/female avatars) in a randomized order. Thus, avatar body type (ABT: muscular/normal) was a between-participant factor, and the gender of the avatar (GoA: male/female) was a within-participant factor. In both virtual conditions, participants wore an HMD and performed the same self-familiarization task as in the first experiment (in VR). However, in the second experiment, participants were given 100 s instead of 60 s in the first experiment. A detailed description of the self-familiarization task is provided in Fig. 2.

### Data analysis

All dependent variables were tested for normality using Kolmogorov-Smirnov or Shapiro-Wilk analysis and measures of skewness and kurtosis, and the assumption of normality was met for all dependent variables. A two-way or three-way analysis of variance (ANOVA) with corrected post hoc tests was performed with one within-participant factor (Environment) and one between-participant factor (ABT) in the first experiment, and one within-participant factor (GoA) and two between-participant factors (ABT, GoP) in the second experiment; $p<0.05$ was considered significant. All data were analyzed using IBM SPSS statistical software version 29 (IBM, Armonk, NY, USA).

## Results

### First experiment

**Figure 5:**
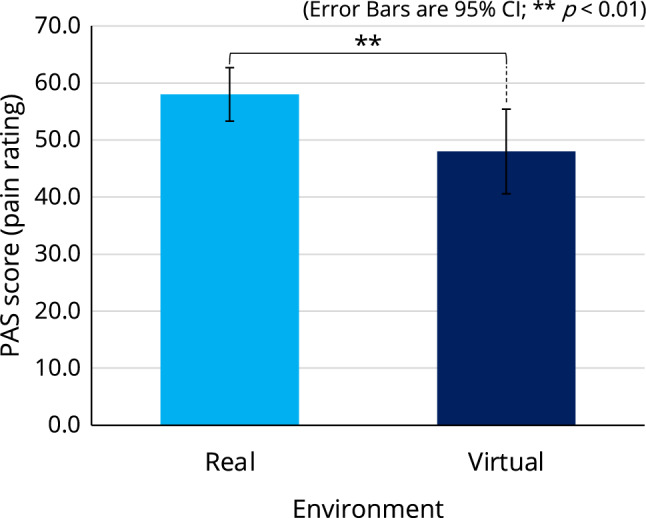
Comparison of PAS scores between the real and virtual environments in the first experiment.

A two-way analysis of variance (ANOVA) with a within-participant factor of environments (real/virtual) and a between-participant factor of avatar body types (ABT: muscular/normal) was conducted to analyze the effect on participants’ PAS scores. While there was no significant interaction between these two factors, a significant main effect of environment was found ($F[1,18]=9.945$, $p<0.01$, $\eta _p^2=0.356$), and PAS scores in the virtual environment (*Mean* : 48.000, *SE* : 3.536) were significantly lower than those in the real environment (*Mean* : 58.000, *SE* : 2.236) (see Fig. 5). However, there was no significant main effect of ABT (see Supplementary Table S4 for all related statistics).

### Second experiment

#### PAS (pain rating)

**Figure 6:**
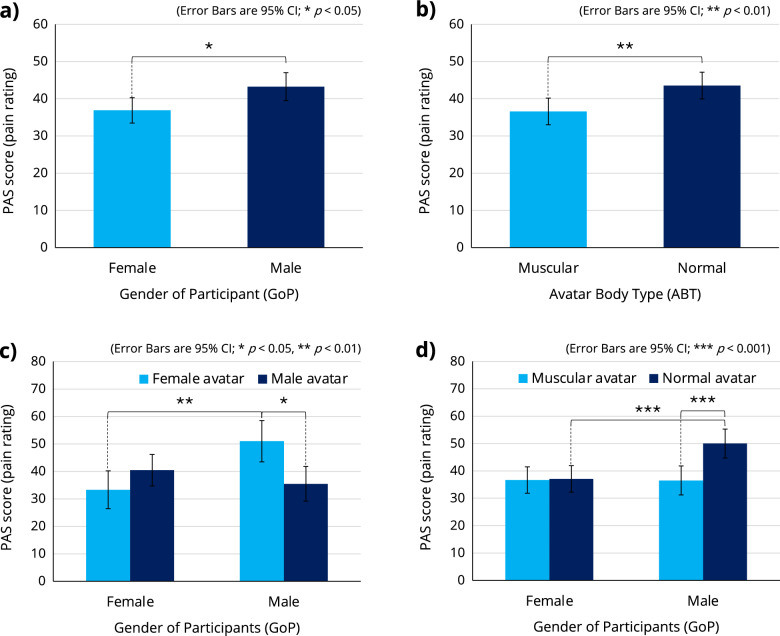
A three-way ANOVA with a within-participant factor of GoA and two between-participant factors of GoP and ABT was performed to analyze their effects on the PAS score in the second experiment. While there was no significant second-order interaction between the three factors, two significant first-order interactions were found: (**c**) GoA$\times$GoP and (**d**) GoP$\times$ABT. There were also two significant main effects of (**a**) GoP and (**b**) ABT. See Supplementary Table S5 for all related statistics.

A three-way ANOVA with a within-participant factor of GoA and two between-participant factors of GoP and ABT was performed to analyze their effects on the PAS score (see Supplementary Table S5 for all related statistics). While there was no significant second-order interaction between the three factors, two significant first-order interactions were found, between GoA and GoP ($F[1,40]=8.316$, $p<0.01$, $\eta _p^2=0.172$) and between GoP and ABT ($F[1,40]=6.810$, $p<0.05$, $\eta _p^2=0.145$). In addition, there were two significant main effects of GoP ($F[1,40]=6.467$, $p<0.05$, $\eta _p^2=0.139$) and ABT ($F[1,40]=7.705$, $p<0.01$, $\eta _p^2=0.162$): the PAS scores of male participants (*Mean* : 43.250, *SE* : 1.851) were significantly higher than those of female participants (*Mean* : 36.875, *SE* : 1.690) (Fig. 6a), and participants reported significantly lower PAS scores when using a muscular avatar (*Mean* : 36.583, *SE* : 1.773) than when using a normal avatar (*Mean* : 43.542, *SE* : 1.773) (Fig. 6b).

Regarding the first-order interaction between GoA and GoP, the Bonferroni post hoc test revealed that male participants reported significantly lower PAS scores when using a male avatar than when using a female avatar ($\Delta \,Mean=15.500$, $SE=5.784$, $p<0.05$), and male participants using a female avatar reported significantly higher PAS scores than female participants using the same female avatar ($\Delta \,Mean=17.667$, $SE=5.040$, $p<0.01$) (see Fig. 6c).

Regarding the first-order interaction between GoP and ABT, the Bonferroni post hoc test revealed that male participants reported significantly lower PAS scores when using a muscular avatar than when using a normal avatar ($\Delta \,Mean=13.500$, $SE=3.703$, $p<0.001$), and female participants using the normal avatar reported significantly lower PAS scores than male participants using the same normal avatar ($\Delta \,Mean=12.917$, $SE=3.545$, $p<0.001$) (see Fig. 6d).

#### GREP (stereotypes about pain)

The GREP, which consists of 15 questions (see Supplementary Table S2), revealed no significant difference between male and female participants in the answers to Q5-Q15. On the other hand, Q1/Q2 (typical woman/man’s sensitivity to pain) and Q3/Q4 (typical muscular/normal body type has a sensitivity to pain) concern our hypothesis.

**Figure 7:**
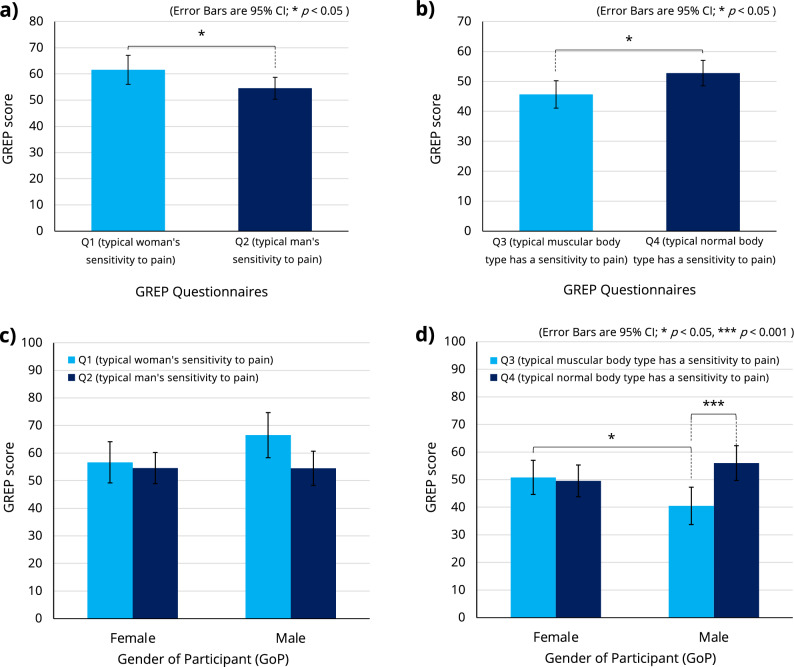
A two-way ANOVA was performed with a between-participant factor of GoP and a within-participant factor of gender (woman/man) in questions (Q1 and Q2) to analyze their effects on the GREP score (see Supplementary Table S6 for all related statistics). The interaction was not significant (**c**); however, a significant main effect of gender in questions was found (**a**). A two-way ANOVA was performed with a between-participant factor of GoP and a within-participant factor of body type (muscular/normal) in questions (Q3 and Q4) to analyze their effects on the GREP score (see Supplementary Table S7 for all related statistics). A significant interaction was found (**d**). A significant main effect of body type was also found (**b**).

For Q1/Q2, a two-way ANOVA was performed with a between-participant factor of GoP and a within-participant factor of gender (woman/man) in the questions to analyze their effects on the GREP score (see Supplementary Table S6 for all related statistics). The interaction was not significant. A significant main effect of gender was found ($F[1,42]=4.657$, $p<0.05$, $\eta _p^2=0.100$) (see Fig. 7a).

For Q3/Q4, a two-way ANOVA was performed with a between-participant factor of GoP and a within-participant factor of body type (muscular/normal) in the questions to analyze their effects on the GREP score (see Supplementary Table S7 for all related statistics). A significant interaction was found ($F[1,42]=8.370$, $p<0.01$, $\eta _p^2=0.166$). A significant main effect of body type was also found ($F[1,42]=6.058$, $p<0.05$, $\eta _p^2=0.126$) (see Fig. 7b). The Bonferroni post hoc test revealed that male participants answered significantly lower pain sensitivity scores to the muscular body type question than to the normal body type question ($\Delta \,Mean=15.500$, $SE=4.276$, $p<0.001$), and male participants answered significantly lower pain sensitivity scores to the muscular body type question than female participants ($\Delta \,Mean=10.333$, $SE=4.548$, $p<0.05$) (see Fig. 7d).

#### The sense of embodiment

**Figure 8:**
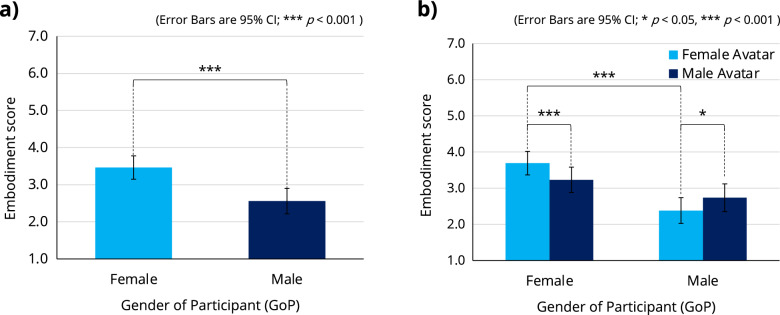
A three-way ANOVA with a within-participant factor of GoA and two between-participant factors of GoP and ABT was performed to analyze their effects on the embodiment score (see Supplementary Table S8 for all related statistics). While there was no significant second-order interaction between the three factors, the interaction between GoA and GoP was significant (**b**). There was also a significant main effect of GoP (**a**).

A three-way ANOVA with a within-participant factor of GoA and two between-participant factors of GoP and ABT was performed to analyze their effects on the embodiment score (see Supplementary Table S8 for all related statistics). While there was no significant second-order interaction between the three factors, the interaction between GoA and GoP ($F[1,40]=19.620$, $p<0.001$, $\eta _p^2=0.329$) was significant. There was also a significant main effect of GoP ($F[1,40]=15.430$, $p<0.001$, $\eta _p^2=0.278$) (see Fig. 8a).

The Bonferroni post hoc test revealed that male participants reported significantly higher embodiment scores when using a male avatar than when using a female avatar ($\Delta \,Mean=0.354$, $SE=0.136$, $p<0.05$), and female participants reported significantly higher embodiment scores when using a female avatar than when using a male avatar ($\Delta \,Mean=0.463$, $SE=0.124$, $p<0.001$) (see Fig. 8b). The difference in embodiment scores between male/female participants when using a female avatar was significant ($\Delta \,Mean=1.313$, $SE=0.239$, $p<0.001$); however, the difference was not significant when using a male avatar (Fig. 8b).

## Discussion

Previous studies have reported that VR can attenuate pain by diverting human attention away from pain perception^5–9^. This effect has been attributed to the immersive environment of VR, which disrupts the conventional focus on pain. Indeed, in our first experiment, although we only used male avatars for male/female participants, the use of these avatars resulted in a 17.241% reduction in participants’ pain perception score (Fig. 5) compared to the real-world baseline condition. This is consistent with existing studies^6–9^ suggesting that immersion in the VR environment can reduce pain perception. Thus, our hypothesis H1 was supported; however, the results of this first experiment were not strong enough to support H2, as we did not find the significant main effect of avatar body type (ABT: muscular/normal). At the same time, we noticed that some female participants had commented on a gender discrepancy in the avatars they used. By conducting this first experiment, we realized the crucial role of gender factors, such as the gender of the participant and the gender of the avatar.

We then conducted the second experiment, which introduced female avatars and carefully controlled for gender factors. The results of the second experiment showed a significant main effect of ABT (using a muscular avatar resulted in a 15.982% reduction in participants’ pain perception score compared to using a normal avatar), supporting H2. The results also revealed two significant first-order interactions: GoA $\times$ GoP and GoP $\times$ ABT, suggesting gender differences in the Proteus effect. In summary, post hoc analyses suggest that (1) the user’s perceived pain intensity is effectively decreased when the user’s gender and the avatar’s gender are congruent, and (2) the use of a muscular avatar is particularly effective for male users. Finally, the second experiment revealed a significant main effect of GoP (male/female), suggesting that female participants (using any avatar in this study) reported less pain than male participants. Female participants’ PAS scores (regardless of avatar type/gender) were actually lower than their baseline scores measured in Phase 1 when not using an avatar, which was not the case for male participants.

Much of the literature on pain research presents evidence that women are more sensitive to pain than men, perceiving relatively low levels of pain more intensely^38–42^. It has also been shown that women’s facial skin contains 34 nerve fibers per square centimeter, while men have only 17, suggesting that this higher density of nerve fibers means enhanced transmission of pain signals, possibly leading to increased pain sensitivity^43^. In addition, the female hormone estrogen is known to lower the pain threshold, the minimal stimulus needed to produce the perception of pain, while the male hormone testosterone can reduce sensitivity to pain^38,41,44^. These biological differences may partially explain the differences in pain perception between the sexes. However, there is also evidence that women quickly habituate to repeated identical stimuli^45^ and experience less pain than men, reflecting that various factors can influence pain perception and response in complex ways. Separately, sociocultural factors such as societal roles, accessibility, and frequency of use of medical services are also known to significantly influence pain perception and sensitivity^42^.

Our study used the GREP questionnaire to examine gender stereotypes of pain among participants and to assess the influence of these stereotypes on pain perception. The results showed that male participants tended to rate women’s pain sensitivity higher (Fig. 7c). This may be related to the higher pain ratings recorded by male participants when using a female avatar (Fig. 6c). In addition, when examining pain perception stereotypes related to body type, the pain sensitivity of muscular figures was rated significantly lower by male participants (Fig. 7d). This is very consistent with the results showing a significant reduction in pain perception when male participants used a muscular avatar, while female participants showed no significant difference in pain perception regardless of ABT (Fig. 6d). These results suggest that one’s own stereotypes could potentially influence the Proteus effect on pain perception. Indeed, stereotypes are known to influence pain in the real world (without the use of VR/avatars)^46^. Investigating the relationship between stereotypes and the Proteus effect in VR is an interesting topic for future research.

The results of the sense of embodiment (Fig. 8b) showed that participants felt significantly more embodied when using a gender-congruent avatar. On the other hand, as discussed above, participants’ perceived pain intensity was decreased when using a gender-congruent avatar (Fig. 6c). Judging from these results, the degree of immersion in an avatar could possibly affect the user’s perception of pain. A relationship between the user’s sense of embodiment and a gender-congruent avatar has been discussed in another case of decision-making^47^.

We also used embodiment scores to assess participants’ level of immersion and their ability to control the avatar. Our results, detailed in Supplementary Table S8, indicate that there was no significant variation in immersion across ABTs. The consistent embodiment scores across avatars suggest that participants experienced a consistent level of immersion and identification with their avatars, regardless of the avatar’s body type. Despite the consistent immersion experience, there was a significant decrease in pain perception associated with the ABT, as shown in Fig. 6b. This consistency in embodiment, coupled with the observed variation in pain perception, suggests that the pain alleviation effect is due to the Proteus effect rather than mere immersion. Our study revealed that specific avatar characteristics significantly influence users’ pain perception, even when embodiment and immersion levels are similar.

Our study has several limitations. First, the demographics of the participants were relatively biased: mostly Chinese students recruited from a university in Japan. Also, we standardized the appearance of the avatars for each gender and designed the avatars without considering the participants’ nationalities. In addition, to maximize the impact of the Proteus effect, we used avatars with muscular physiques, which are uncommon in reality. Previous research^48^ has shown that participants did not internalize the physical changes induced by visual illusions, resulting in a lack of immersion and no observed improvement in skills. This underscores the importance of internalization in achieving the intended effects of such interventions. Future research should include a process for participants to select their own avatars prior to the experiments. This approach would not only allow for more personalized avatar design, but also allow for comprehensive studies that account for a wide range of pain types and individual differences in pain perception and response. Recent studies have highlighted the importance of considering user diversity in avatar design. For example, research on how different avatar hands are perceived differently depending on the gender of the user supports the need for avatar selection processes that reflect individual identities and preferences^49^. This approach could help deepen our understanding of the impact of avatar selection on the Proteus effect and pain perception.

This study highlights the interplay of identity and physicality in the perception of acute pain when presented to healthy adults. The findings not only advance our understanding of pain, but also open avenues for innovative pain management strategies using VR.

## Supplementary Information

Supplementary Information.

## Data Availability

The datasets used and/or analysed during the current study available from the corresponding author on reasonable request.
