# Supplementary material for: The proteus effect on human pain perception through avatar muscularity and gender factors
Source: Sci Rep. 2024 May 23;14:11332. doi: 10.1038/s41598-024-61409-4 (PMC11632092; doi:10.1038/s41598-024-61409-4)
Supplement: Supplementary file 1 — Supplementary Information. [file 41598_2024_61409_MOESM1_ESM.pdf]

The Proteus Effect on Human Pain Perception Through Avatar Muscularity and Gender Factors – Supplementary Information –

Youchan Yim[1], Zongheng Xia[1], Yuki Kubota[1], and Fumihide Tanaka[1,*]

[1]University of Tsukuba, Tsukuba, 305-8573, Japan
[*]fumihide.tanaka@gmail.com

This supplementary information includes:

Figure section

- Figure S1 : PAS for rating pain during the experiment.

Table section

- Table S1 : The distribution of participants.
- Table S2 : The modified GREP question items and t-test results.
- Table S3 : The Sense of Embodiment Questionnaire.
- Table S4 : Results of a two-way repeated measures ANOVA (Environment×ABT) on the PAS score.
- Table S5 : Results of a three-way repeated measures ANOVA (GoA×GoP×ABT) on the PAS score.
- Table S6 : Results of a two-way repeated measures ANOVA (GoP×gender factor) on the GREP score.
- Table S7 : Results of a two-way repeated measures ANOVA (GoP×body type factor) on the GREP score.
- Table S8 : Results of a three-way repeated measures ANOVA (GoA×GoP×ABT) on the Embodiment score.

Supplementary Figures

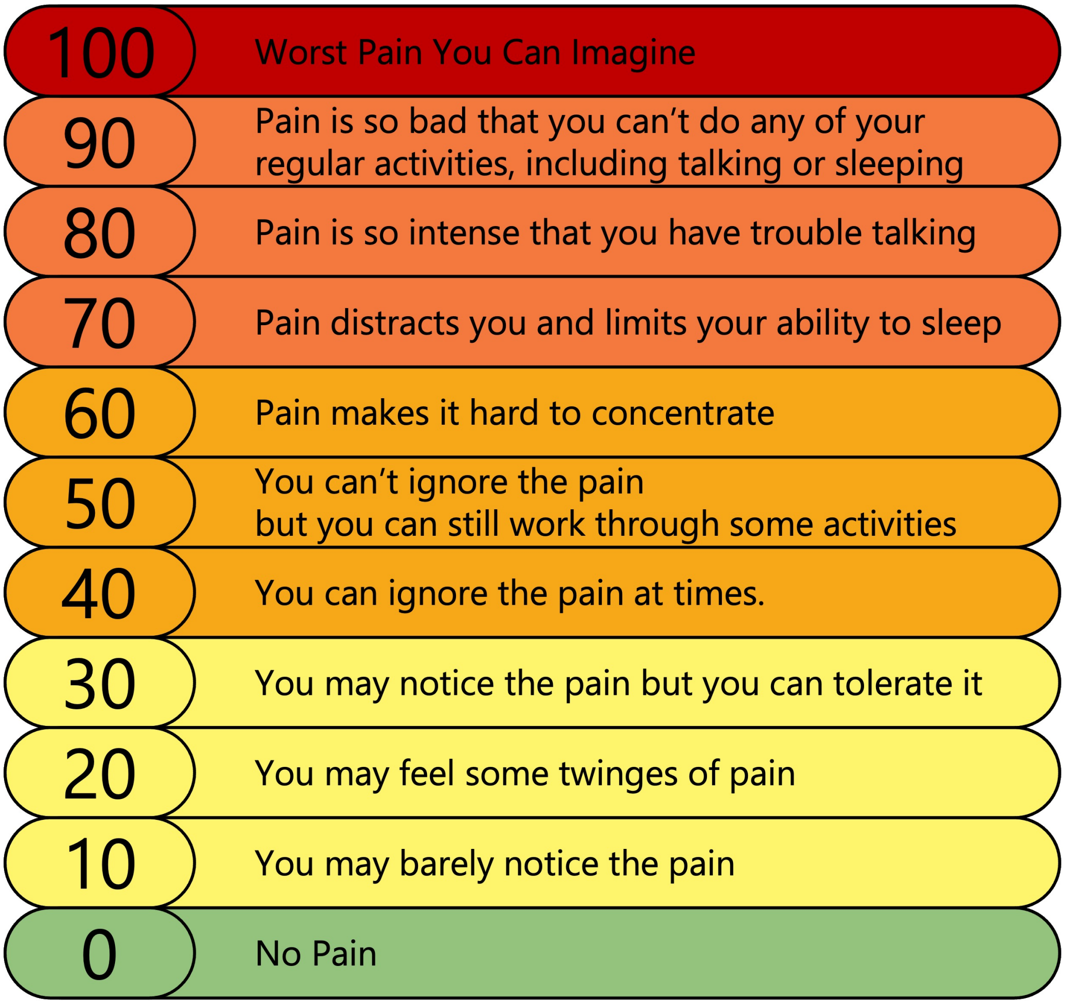

Figure. S1. PAS for rating pain during the experiment.

Supplementary Tables

Table S1. The distribution of participants.

1. First Experiment

ABT	GoP	N	Mean age (SD)
Muscular	Female	5	26.400 (4.630)
	Male	5	25.000 (1.789)
Normal	Female	5	26.800 (1.600)
	Male	5	24.800(1.600)
**Total**		**20**	**25.750 (2.861)**

2. Second Experiment

ABT	GoP	N	Mean age (SD)
Muscular	Female	12	25.000 (1.826)
	Male	10	24.900 (1.375)
Normal	Female	12	24.167 (2.115)
	Male	10	26.100 (2.166)
**Total**		**44**	**25.000 (2.023)**

Table S2. The modified GREP question items and t-test results.

Domain	No.	Question item	Female mean (SD)	Male mean (SD)	t-value	df	p-value	Δ Mean	SE
	Q1	Typical woman's sensitivity to pain	56.667 (19.035)	66.500 (16.944)	-1.793	42	0.080	9.833	5.486
	Q2	Typical man's sensitivity to pain	54.583 (13.507)	54.500 (13.945)	0.020	42	0.984	0.083	4.150
Sensitivity	Q3	Typical muscular body type has a sensitivity to pain	50.833 (17.425)	40.500 (11.459)	2.357	42.020	0.023	10.333	4.384
	Q4	Typical normal body type has a sensitivity to pain	49.583 (14.885)	56.000 (12.732)	-1.519	42	0.136	6.417	4.224
	Q5	Your sensitivity to pain	53.333 (14.646)	52.000 (16.733)	0.282	42	0.779	1.333	4.731
	Q6	Typical woman's pain endurance	59.167 (22.634)	60.000 (22.243)	-0.123	42	0.903	0.833	6.800
	Q7	Typical man's pain endurance	55.000 (19.781)	63.000 (15.252)	-1.478	42	0.147	8.000	5.412
Endurance	Q8	Typical muscular body type's pain endurance	67.500 (17.754)	72.500 (16.182)	-0.968	42	0.339	5.000	5.165
	Q9	Typical normal body type's pain endurance	54.167 (16.918)	60.000 (11.698)	-1.303	42	0.200	5.833	4.477
	Q10	Your pain endurance	56.250 (20.176)	64.000 (16.351)	-1.380	42	0.175	7.750	5.614
	Q11	Typical woman's willingness to report pain	69.167 (15.299)	73.000 (18.382)	-0.755	42	0.454	3.833	5.076
	Q12	Typical man's willingness to report pain	40.833 (19.318)	47.500 (17.130)	-1.199	42	0.237	6.667	5.559
Willingness	Q13	Typical muscular body type's willingness to report pain	36.667 (17.611)	43.500 (22.070)	-1.143	42	0.260	6.833	5.981
	Q14	Typical normal body type's willingness to report pain	52.500 (15.108)	54.500 (12.763)	-0.469	42	0.642	2.000	4.268
	Q15	Your willingness to report pain	54.583 (25.363)	55.000 (23.056)	-0.057	42	0.955	0.417	7.371

Table S3. The Sense of Embodiment Questionnaire.

No.	Question item
Q1	I felt out of my body
Q2	I felt as if my (real) body were drifting toward the virtual body or as if the virtual body were drifting toward my (real) body
Q3	I felt as if the movements of the virtual body were influencing my own movements
Q4	It felt as if my (real) body were turning into an 'avatar' body
Q5	At some point it felt as if my real body was starting to take on the posture or shape of the virtual body that I saw
Q6	I felt like I was wearing different clothes from when I came to the laboratory
Q7	I felt as if my body had changed
Q8	I felt a stronger sensation in my body when I saw this avatar
Q9	I felt that my own body could be affected by avatar's body shape
Q10	I felt as if the virtual body was my body
Q11	At some point it felt that the virtual body resembled my own (real) body, in terms of shape, skin tone or other visual features
Q12	I felt as if my body was located where I saw the virtual body
Q13	I felt like I could control the virtual body as if it was my own body
**Appearance**	(Q1 + Q2 + Q3 + Q4 + Q5 + Q6 + Q9) / 7
**Response**	(Q4 + Q6 + Q7 + Q8 + Q9) / 5
**Ownership**	(Q5 + Q10 + Q11 + Q12 + Q13) / 5
**Multi-Sensory**	(Q3 + Q12 + Q13) / 3
**Embodiment**	(**Appearance** + **Response** + **Ownership** + **Multi-Sensory**) / 4

Table S4

1. Results of a two-way repeated measures ANOVA (Environment×ABT) on the PAS score.

Effect	F value	Hyp. df	Error df	p-value	η_p^2
Environment	9.945	1	18	0.005	0.356
ABT	1.002	1	18	0.330	0.053
Environment×ABT	0.895	1	18	0.357	0.047

2. Results of Bonferroni post hoc comparisons (main effect : Environment).

Real (SE)	Virtual (SE)	Δ Mean	SE	p-value
58.000 (2.236)	48.000 (3.536)	10.000	3.171	0.005

Table S5

1. Results of a three-way repeated measures ANOVA (GoA×GoP×ABT) on the PAS score.

Effect	F value	Hyp. df	Error df	p-value	η_p^2
GoA	1.155	1	40	0.289	0.028
GoP	6.467	1	40	0.015	0.139
ABT	7.705	1	40	0.008	0.162
GoA×GoP	8.316	1	40	0.006	0.172
GoA×ABT	0.139	1	40	0.712	0.003
GoP×ABT	6.810	1	40	0.013	0.145
GoA×GoP×ABT	1.022	1	40	0.318	0.025

2. Results of Bonferroni post hoc comparisons (interaction : GoA×GoP).

GoA \ GoP	Female	Male	Δ Mean	SE	p-value
Female avatar	33.333	51.000	17.667	5.040	0.001
Male avatar	40.417	35.500	4.917	4.223	0.251

GoP \ GoA	Female avatar	Male avatar	Δ Mean	SE	p-value
Female	33.333	40.417	7.083	5.280	0.187
Male	51.000	35.500	15.500	5.784	0.011

3. Results of Bonferroni post hoc comparisons (interaction : GoP×ABT).

ABT \ GoP	Female	Male	Δ Mean	SE	p-value
Muscular	36.667	36.500	0.167	3.545	0.963
Normal	37.083	50.000	12.917	3.545	< 0.001

GoP \ ABT	Muscular	Normal	Δ Mean	SE	p-value
Female	36.667	37.083	0.417	3.380	0.903
Male	36.500	50.000	13.500	3.703	< 0.001

4. Results of Bonferroni post hoc comparisons (main effects : GoP and ABT)

Main effect	Level	Mean (SE)	Δ Mean	SE	p-value
GoP	Female	36.875 (1.690)	6.375	2.507	0.015
	Male	43.250 (1.851)			
ABT	Muscular	36.583 (1.773)	6.958	2.507	0.008
	Normal	43.542 (1.773)			

Table S6

1. Results of a two-way repeated measures ANOVA (GoP×gender factor) on the GREP score.

Effect	F value	Hyp. df	Error df	p-value	η_p^2
Sensitivity					
GoP	1.827	1	42	0.184	0.042
gender factor in Q1-Q2	4.657	1	42	0.037	0.100
GoP×gender factor in Q1-Q2	2.309	1	42	0.136	0.052
Endurance					
Effect	F value	Hyp. df	Error df	p-value	η_p^2
GoP	0.907	1	42	0.346	0.021
gender factor in Q6-Q7	0.021	1	42	0.886	0.000
GoP×gender factor in Q6-Q7	0.790	1	42	0.379	0.018
Willingness					
Effect	F value	Hyp. df	Error df	p-value	η_p^2
GoP	1.892	1	42	0.176	0.043
gender factor in Q11-Q12	52.632	1	42	< 0.001	0.556
GoP×gender factor in Q11-Q12	0.146	1	42	0.705	0.003

2. Results of Bonferroni post hoc comparisons for GREP scores (main effects)

Domain	No.	Mean (SE)	Δ Mean	SE	p-value
Sensitivity	Q1	61.583 (2.743)	7.042	3.263	0.037
	Q2	54.542 (2.075)			
Willingness	Q11	71.083 (2.538)	26.917	3.710	< 0.001
	Q12	44.167 (2.779)			

Table S7

1. Results of a two-way repeated measures ANOVA (GoP×body type factor) on the GREP score.

Sensitivity					
Effect	F value	Hyp. df	Error df	p-value	η_p^2
GoP	0.352	1	42	0.556	0.008
body type factor in Q3-Q4	6.058	1	42	0.018	0.126
GoP×body type factor in Q3-Q4	8.370	1	42	0.006	0.166
**Endurance**					
Effect	F value	Hyp. df	Error df	p-value	η_p^2
GoP	1.774	1	42	0.190	0.041
body type factor in Q8-Q9	24.446	1	42	< 0.001	0.368
GoP×body type factor in Q8-Q9	0.025	1	42	0.874	0.001
**Willingness**					
Effect	F value	Hyp. df	Error df	p-value	η_p^2
GoP	0.919	1	42	0.343	0.021
body type factor in Q13-Q14	31.231	1	42	< 0.001	0.426
GoP×body type factor in Q13-Q14	1.013	1	42	0.320	0.024

2. Results of Bonferroni post hoc comparisons for Sensitivity GREP scores (interaction : GoP×body type factor in Q3-Q4).

GoP \ body type	Q3	Q4	Δ Mean	SE	p-value
Female	50.833	49.583	1.250	3.903	0.750
Male	40.500	56.000	15.500	4.276	< 0.001

body type \ GoP	Female	Male	Δ Mean	SE	p-value
Q3	50.833	40.500	10.333	4.548	0.028
Q4	49.583	56.000	6.417	4.224	0.136

3. Results of Bonferroni post hoc comparisons for GREP scores (main effects)

Domain	No.	Mean (SE)	Δ Mean	SE	p-value
Sensitivity	Q3	45.667 (2.274)	7.125	2.895	0.018
	Q4	52.792 (2.112)			
Endurance	Q8	70.000 (2.583)	12.917	2.612	< 0.001
	Q9	57.083 (2.238)			
Willingness	Q13	40.083 (2.990)	13.417	2.401	< 0.001
	Q14	53.500 (2.134)			

Table S8

1. Results of a three-way repeated measures ANOVA (GoA×GoP×ABT) on the Embodiment score.

Effect	F value	Hyp. df	Error df	p-value	η_p^2
GoA	0.350	1	40	0.558	0.009
GoP	15.430	1	40	< 0.001	0.278
ABT	0.246	1	40	0.622	0.006
GoA×GoP	19.620	1	40	< 0.001	0.329
GoA×ABT	1.503	1	40	0.227	0.036
GoP×ABT	1.749	1	40	0.194	0.042
GoA×GoP×ABT	0.362	1	40	0.551	0.009

2. Results of Bonferroni post hoc comparisons (interaction : GoA×GoP).

GoA \ GoP	Female	Male	Δ Mean	SE	p-value
Female avatar	3.694	2.381	1.313	0.239	< 0.001
Male avatar	3.232	2.735	0.497	0.257	0.061

GoP \ GoA	Female avatar	Male avatar	Δ Mean	SE	p-value
Female	3.694	3.232	0.463	0.124	< 0.001
Male	2.381	2.735	0.354	0.136	0.013

3. Results of Bonferroni post hoc comparisons (main effect : GoP)

Main effect	Level	Mean (SE)	Δ Mean	SE	p-value
GoP	Female	3.463 (0.155)	0.905	0.230	< 0.001
	Male	2.558 (0.170)			
